# ﻿Bees of subfamily Nomiinae (Hymenoptera, Halictidae) from Southern Punjab, Pakistan

**DOI:** 10.3897/zookeys.1238.139993

**Published:** 2025-05-19

**Authors:** Huanhuan Chen, Waseem Akram, Muhammad Naeem, Nawaz Haider Bashir, Sabir Hussain, Maryam Riasat, Asif Sajjad, Ruilin Tian, Ammad Ahmad, Muhammad Khalid Rafique, Zamin Hussain Dahri

**Affiliations:** 1 College of Biological Resources and Food Engineering, Qujing Normal University, Qujing 655011, Yunnan, China Qujing Normal University Qujing China; 2 Honeybee Research Institute (HBRI), National Agricultural Research Centre (NARC), Islamabad, Pakistan The Islamia University of Bahawalpur Bahawalpur Pakistan; 3 Department of Entomology, The Islamia University of Bahawalpur, Bahawalpur, 63100, Pakistan Honeybee Research Institute (HBRI), National Agriculture Research Center (NARC) Islamabad Pakistan; 4 Department of Zoology, Faculty of Engineering and Applied Sciences, Riphah International University, Faisalabad Campus, Faisalabad, 38000, Pakistan Riphah International University Faisalabad Pakistan; 5 Key Laboratory of Biodiversity Conservation and Sustainable Utilization for College and University of Inner Mongolia Autonomous Region, College of Life Science and Technology, Inner Mongolia Normal University, Hohhot 010022, China Inner Mongolia Normal University Hohhot China; 6 Beekeeping and Hill Fruit Pest Research Station, Murree, Pakistan Beekeeping and Hill Fruit Pest Research Station Murree Pakistan

**Keywords:** *Austronomia*, checklist, keys, *Lipotriches*, *Nomia*, *Pseudapis*, taxonomy

## Abstract

To date, 26 species of the subfamily Nomiinae are known to occur in Pakistan. Among these, most of the species have been reported from the northern parts of Punjab, particularly the Pothwar region. In this study, sweat bees from the subfamily Nomiinae were collected from six districts of southern Punjab, Pakistan, to identify their taxonomic diversity. A total of nine species from four genera i.e., *Austronomia* Michener, 1965, *Lipotriches* Gerstaecker, 1858, *Nomia* Letreille, 1804, and *Pseudapis* Kirby, 1900 were identified. These species were *Austronomiapilipes* (Smith, 1875), Lipotriches (Armatriches) fervida (Smith, 1875), L. (Lipotriches) fulvinerva (Cameron, 1907), Nomia (Leuconomia) interstitialis Cameron, 1898, N. (Nomia) curvipes (Fabricius, 1793), N. (Hoplonomia) westwoodi (Gribodo, 1894), Pseudapis (Pseudapis) nilotica (Smith, 1875), P. (Pseudapis) oxybeloides (Smith, 1875), and P. (Nomiapis) bispinosa (Brulle, 1832). All the bee species except *P.oxybeloides* are reported for the first time from southern Punjab, Pakistan. A key to the genera of the subfamily Nomiinae, species, diagnoses, floral hosts, and distributions are provided. Moreover, habitus photographs and male genitalia illustrations are provided for each species except *L.fervida* and *L.fulvinerva*, as no male specimens were collected during the entire study period. This study will be helpful in the establishment of conservation strategies for native bees.

## ﻿Introduction

Insects, particularly bees, provide the most important ecosystem service, leading to an increase in quality and quantity (Potts et al. 2010) of approximately 35% of crop production globally (Klein et al. 2007). The bees originated approximately 120 million years ago and are considered an ancient group of insects (Roubik 1992). Bees have been traditionally involved in human civilization, especially in agriculture and rural economies (Combey 2008). There are more than 20,000 bee species found throughout the world (Michener 2007), and among these solitary bees are more abundant that neither produce honey nor other by-products nor live in hives but provide pollination services as pollinating agents of wild and cultivated plants (Luck et al. 2009; Gallai et al. 2009). A few features make them more efficient pollinators than others, i.e., the scopa or special hairs on their body for carrying pollen grains (Karunaratne and Edirisinghe 2006; Sajjad et al. 2019a, Akram et al. 2022; Akram and Sajjad 2022) and the ability of sonication or buzz pollination (Abrol 2015; Russell et al. 2016). Most of the pollination studies have found that these solitary bees are equal to or higher in efficiency than the domesticated honey bee (Kremen et al. 2002; Winfree et al. 2007; Mayes 2011; Breeze et al. 2011; Ollerton et al. 2011; Hogendoorn and Keller 2012; Rader et al. 2012; Garibaldi et al. 2014). Vaughan et al. (2007) assessed the value of ecosystem services, especially pollination provided by the native bees (non-*Apis*) in North America alone, to be ~ 3 billion US dollars.

Bees of the superfamily Apoidea are classified into seven families, i.e., Apidae, Andrenidae, Colletidae, Halictidae, Megachilidae, Melittidae, and Stenotritidae (Michener 2000, 2007). Among these, Apidae is considered the largest bee family, containing ~ 6184 described species, followed by Halictidae, which includes more than 4494 species throughout the world (Ascher and Pickering 2024). The subfamily Nomiinae consists of 17 genera with 626 reported species throughout the world (Ascher and Pickering 2024). From Pakistan, ~ 335 bee species have been reported so far and among them, 26 species are from the subfamily Nomiinae (Ascher and Rasmussen 2010; Ascher and Pickering 2024).

Almost all of the literature regarding the taxonomic identity of Nomiinae bees is documented only for the genus *Nomia* from the Potohar region of Pakistan (Bodlah et al. 2016; Aslam et al. 2020; Bodlah et al. 2020). No literature is documented about the bees of the subfamily Nomiinae from southern Punjab, Pakistan. Therefore, this study aimed to assess the taxonomic identity of Nomiinae bee genera i.e., *Austronomia*, *Lipotriches*, *Nomia*, and *Pseudapis* from southern Punjab, Pakistan.

## ﻿Materials and methods

**Figure 1.**
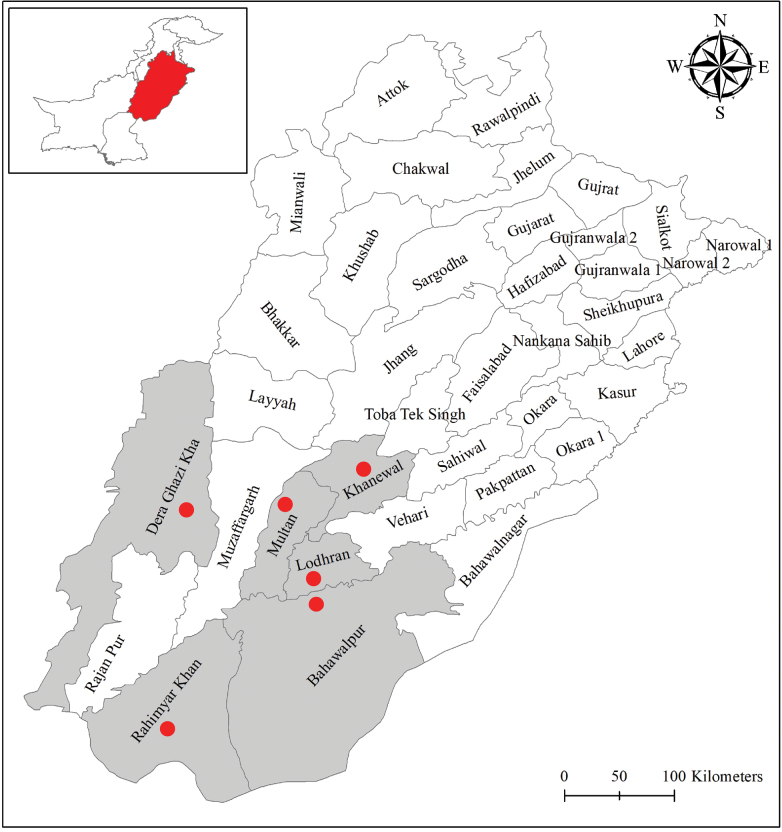
Map of Punjab Province reflecting the major districts of southern Punjab. The districts highlighted in grey are the collection sites (collected randomly for three consecutive days from various land use types, i.e., desert, forest, public parks, alongside roads, and agricultural fields). Red circle points indicate the collection sites.

The study was carried out in six districts of southern Punjab, i.e., Bahawalpur (29°24'33"N, 71°39'50"E), Lodhran (29°31'58"N, 71°37'51"E), Rahimyar Khan (28°25'1"N, 70°18'15"E), Khanewal (30°18'53"N, 72°2'2"E), Multan (30°11'24"N, 71°27'28"E), and Dera Ghazi Khan (30°2'56"N, 70°37'1"E) (Fig. 1). Geographically, south Punjab is situated in the center of the Pakistan consists of 11 districts divided in three divisions, Bahawalpur, Dera Ghazi Khan, and Multan with a total area of 102,301 km^2^ (Primary & Secondary Healthcare Department 2023). The area experiences a sub-tropical arid climate, characterized by a short cold winter and a long hot summer. There are four different seasons in this region, autumn from September to November, winter from December to February, spring from March to May, and summer from June to August. The spring season witnesses ~ 60% of the plant species blossoms (Sajjad and Saeed 2010, 2012). The region has a variety of bee-supporting landscapes, including agricultural fields, deserts, hill torrents, and artificial forests. The natural vegetation includes desertic herbs, shrubs, and trees.

Nomiinae bees were collected from the first week of October 2019 to the fourth week of September 2021. Sampling was done fortnightly on three consecutive days in the desert, forest, and public parks, alongside roads, and in agricultural fields. Clear and sunny days were selected and rainy and cloudy days were avoided for the sampling. During each sampling day, a random walk was carried out from 0800 hr to 1200 hr, focusing on all the available plant species in flower. A professional entomological aerial net was used to collect specimens as it is an easy and efficient method (Dafni 1992). The bees were immediately killed using ethyl acetate fumes and placed in separate plastic pouches with appropriate labels indicating the location, date, host plant, and collector name, and then shifted to the laboratory for further processing.

In the laboratory, the specimens were properly pinned and labeled with ~3 entomological pins. The first label of each specimen contained information regarding location, date, host plant, and collector name, while the second label contained information regarding taxonomic identity. Pinned specimens were placed in the bee collection drawers.

After pinning, the male specimens became hard, therefore the male specimens of species for genitalia extraction were placed in the relaxing chamber for 24 hours, and then the abdomen of the specimen was removed and placed in petri dishes with proper tagging. A solution of 10% KOH was prepared and poured into a petri dish. The abdomen was kept for 24 hours in the solution to soften the body parts. Genitalia were removed under the stereomicroscope (IRMECO, IM-SZ-500, Schwarzenbek, Germany). The skeletal parts were separated using a pair of sharp needles and fine forceps. The dissected genitalia were preserved in small glass vials containing 70% ethanol.

Bees were identified up to the family, subfamily, tribe, genus, and subgenus level by using the keys provided by Michener (2007). Species were identified using the most relevant published literature (Bingham 1897; Pauly 2008, 2009; Bodlah et al. 2016, 2020; Majumder et al. 2020). Species-level keys for each genus were prepared. Specimens were deposited to the Entomology Laboratory, Faculty of Agriculture and Environment (FA&E), The Islamia University of Bahawalpur (**IUB**). Plants visited by the bees were identified by the local experts (see Acknowledgments).

Each specimen was photographed from the dorsal and lateral sides using a digital camera (Canon 600D) mounted with a 100 mm macro lens. The photographs were edited with the help of Adobe Photoshop 2021.

## ﻿Results and discussion

### ﻿Checklist of previously reported Nomiinae bees

Reference: Ascher and Pickering (2024)

**Genus *Lipotriches* Gerstaecker, 1858**

**Lipotriches (Armatriches) fervida (Smith, 1875)**

**Global distribution.** India and Pakistan.

**Regional distribution.** Sindh.

**Lipotriches (Lipotriches) fulvinerva (Cameron, 1907)**

**Global distribution.** India, Myanmar, and Pakistan.

**Regional distribution.** Punjab and Sindh.

**Lipotriches (Rhopalomelissa) parca (Kohl, 1906)**

**Global distribution.** Egypt, Libya, Niger, Pakistan, Saudi Arabia, Sudan, United Arab Emirates, and Yemen.

**Regional distribution.** Punjab and Sindh.

**Lipotriches (Rhopalomelissa) parcella (Cockerell, 1911)**

**Global distribution.** Pakistan.

**Regional distribution.** Sindh.

**Genus *Austronomia* Michener, 1965**

***Austronomiapilipes* (Smith, 1875)**

**Global distribution.** Pakistan.

**Regional distribution.** Punjab.

**Genus *Macronomia* Cockerell, 1917**

***Macronomiaperlucida* (Cockerell, 1911)**

**Global distribution.** Pakistan.

**Regional distribution.** Sindh.

**Genus *Nomia* Letreille, 1804**

**Nomia (Crocisaspidia) buddha Westwood, 1875**

**Global distribution.** India and Pakistan.

**Nomia (Crocisaspidia) callichlora Cockerell, 1911**

**Global distribution.** India and Pakistan.

**Regional distribution.** Punjab and Sindh.

**Nomia (Nomia) crassipes (Fabricius, 1798)**

**Global distribution.** Bhutan, China, India, Nepal, Pakistan, Sri Lanka, Taiwan, and Thailand.

**Regional distribution.** Khyber Pakhtunkhwa.

**Nomia (Nomia) curvipes (Fabricius, 1793)**

**Global distribution.** India and Pakistan.

**Regional distribution.** Punjab and Sindh.

**Nomia (Leuconomia) interstitialis Cameron, 1898**

**Global distribution.** India, Pakistan, and Sri Lanka.

**Regional distribution.** Punjab.

**Nomia (Crocisaspidia) vespoides Walker, 1871**

**Global distribution.** Eritrea, Iran, Oman, Pakistan, and Sudan.

**Nomia (Hoplonomia) westwoodi Gribodo, 1894**

**Global distribution.** Afghanistan, India, Pakistan, and Sri Lanka.

**Regional distribution.** Sindh (Karachi).

**Genus *Pseudapis* Kirby, 1900**

**Pseudapis (Nomiapis) bispinosa (Brullé, 1832)**

**Global distribution.** Afghanistan, Algeria, Egypt, India, Iran, Iraq, Italy, China, Cyprus, Georgia, Greece, Hungary, Kazakhstan, Malta, Morocco, Pakistan, Russia, Spain, Tunisia, Turkey, Turkmenistan, and Ukraine.

**Regional distribution.** Punjab (Rawalpindi).

**Pseudapis (Nomiapis) diversipes (Latreille, 1806)**

**Global distribution.** Armenia, Azerbaijan, China, Congo, Cyprus, France, Greece, Iran, Italy, Kazakhstan, Kyrgyztan, Macedonia, Mongolia, Pakistan, Poland, Romania, Russia, Slovakia, Spain, Switzerland, Syria, Tajikistan, Turkey, Turkmenistan, Ukraine, and Zimbabwe.

**Regional distribution.** Baluchistan (Quetta) and Punjab (Rawalpindi).

**Pseudapis (Pseudapis) edentata (Morawitz, 1876)**

**Global distribution.** Azerbaijan, India, Iran, Iraq, Oman, Pakistan, Saudi Arabia, Tajikistan, Turkey, Turkmenistan, and Uzbekistan.

**Regional distribution.** Baluchistan.

**Pseudapis (Pseudapis) enecta (Cockerell, 1911)**

**Global distribution.** Pakistan.

**Regional distribution.** Sindh.

**Pseudapis (Pseudapis) flavolobata (Cockerell, 1911)**

**Global distribution.** India, Iran, Mauritania, Pakistan, United Arab Emirates.

**Regional distribution.** Sindh (Karachi).

**Pseudapis (Nomiapis) fugax (Morawitz, 1877)**

**Global distribution.** China, Iran, Kazakhstan, Pakistan, Russia, Turkmenistan, and Uzbekistan.

**Regional distribution.** Baluchistan.

**Pseudapis (Pseudapis) inermis (Morawitz, 1894)**

**Global distribution.** Georgia, Iran, Israel, Pakistan, Saudi Arabia, Tajikistan, Turkmenistan, United Arab Emirates, and Yemen.

**Regional distribution.** Baluchistan (Quetta).

**Pseudapis (Pseudapis) nilotica (Smith, 1875)**

**Global distribution.** Afghanistan, Algeria, Djibouti, Egypt, Ethiopia, Iran, Iraq, Jordan, Libya, Morocco, Niger, Pakistan, Russia, Saudi Arabia, Sudan, Turkmenistan, and United Arab Emirates.

**Regional distribution.** Baluchistan (Quetta) and Sindh (Karachi).

**Pseudapis (Pseudapis) oxybeloides (Smith, 1875)**

**Global distribution.** Bangladesh, India, Pakistan, Sri Lanka, and Russia.

**Regional distribution.** Sindh (Gharo).

**Pseudapis (Nomiapis) squamata (Morawitz, 1894)**

**Global distribution.** Iran, Kazakhstan, Kyrgyzstan, Pakistan, Turkmenistan, and Uzbekistan.

**Regional distribution.** Baluchistan (Quetta).

**Pseudapis (Pseudapis) stenotarsus Baker, 2002**

**Global distribution.** Pakistan and United Arab Emirates.

**Regional distribution.** Khyber Pakhtunkhwa (Mardan).

**Pseudapis (Nomiapis) valga (Gerstäcker, 1872)**

**Global distribution.** Armenia, Azerbaijan, Congo, Cyprus, Greece, Iran, Kazakhstan, Pakistan, Spain, Tajikistan, Turkey, and Turkmenistan.

**Regional distribution.** Baluchistan (Quetta).

**Genus *Steganomus* Ritsema, 1873**

***Steganomusbipunctatus* (Fabricius, 1804)**

**Global distribution.** India, Pakistan, and Sri Lanka.

**Regional distribution.** Khyber Pakhtunkhwa.

### ﻿Nomiinae bees from south Punjab

A total of nine species from the genera *Austronomia*, *Lipotriches*, *Nomia*, and *Pseudapis* are newly recorded from southern Punjab, i.e., *Austronomiapilipes*, *Lipotrichesfervida*, *L.fulvinerva*, *Nomiainterstitialis*, *N.curvipes*, *N.westwoodi*, *Pseudapisnilotica*, *P.oxybeloides* and *P.bispinosa*, were identified and reported from southern Punjab, Pakistan.

### ﻿Key to the genera of subfamily Nomiinae

**Table:** 

1	Terga with integumentary apical bands, metanotum without double projection, tergum 1 with an enameled band, females with incomplete basal plateau of hind tibiae, male with a tooth under the femur	***Nomia***
–	Terga without integumentary apical bands (only sometimes pubescent bands), tergum 1 without an enameled band, females with complete basal plateau of the hind tibiae, male without a tooth under the femur	**2**
2	Tegulae strongly developed, the posterior border reaching the apical margin of the scutum, presence of a subocular keel, terga with pubescent apical bands	***Pseudapis***
–	Tegulae smaller and normal in size	**3**
3	Pronotum with a keel along the entire anterior edge, female with incomplete and linear basal plateau of the posterior tibia	***Lipotriches***
–	Pronotum without a keel on the anterior edge or if present then largely interrupted, females with the basal plateau of the hind tibia keeled or not	***Austronomia***

### ﻿Key to species of *Austronomia* Michener, 1965 and *Lipotriches* Gerstaecker, 1858

**Table:** 

1	Body length range 10–12 mm; mesosoma dorsally covered with yellowish brown hairs (Fig. 2A); tergum 1 with snow-white pubescence (Fig. 2B)	***A.pilipes***
–	Body length 6–7 mm; mesosoma not covered with yellowish brown hairs; tergum 1 without snow-white pubescence	**2**
2	Marginal cell in the forewings fully rounded at the apex (Fig. 2C); tibiae black and tarsi orange (Fig. 2D); metasoma coarsely punctured, not shiny, the apex of terga 1 and 2 medially with less pale layer (Fig. 2E)	***L.fervida***
–	Marginal cell in the forewings not fully rounded at the apex (Fig. 2F); tibiae and tarsi completely orange (Fig. 2G); metasoma not coarsely punctured but shiny, the apex of terga 1 and 2 medially with shiny pale layer (Fig. 2H)	***L.fulvinerva***

#### ﻿ Lipotriches (Armatriches) fervida

Taxon classificationAnimaliaHymenopteraHalictidae

(Smith, 1875)

794882AF-D51B-50DF-8712-606236ACA09D

Figs 2C–E , 3

Nomia fervida Smith, 1875.

##### Material examined.

Pakistan. Punjab, Bahawalpur • 1♀, 8 November 2020, W. Akram.

**Figure 2.**
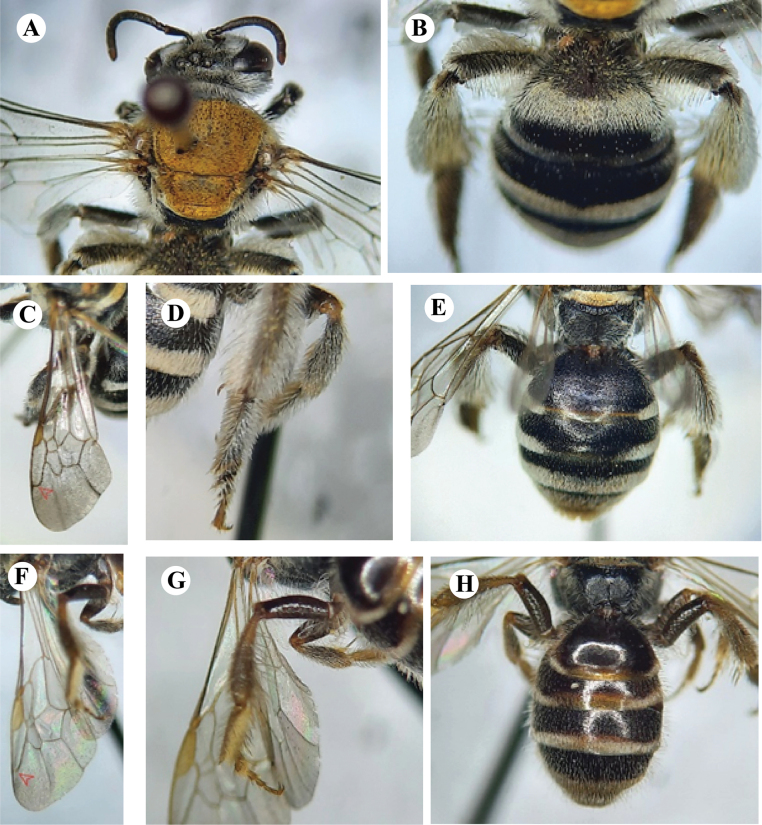
**A, B***Austronomiapilipes* (Smith, 1875) **A** head and mesosoma **B** metasoma showing tergum 1 and hind legs **C–E***Lipotrichesfervida* (Smith, 1875) **C** left forewing showing the marginal cell **D** hind leg **E** metasoma in dorsal view **F–H***L.fulvinerva* (Cameron, 1907) **F** left forewing showing the marginal cell **G** hind leg **H** metasoma in dorsal view.

##### Diagnosis.

**Figure 3.**
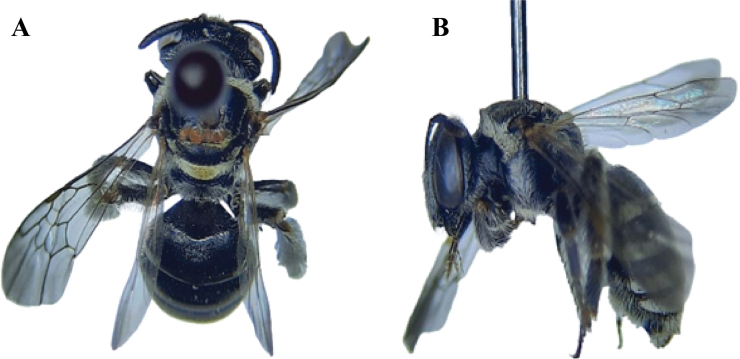
*Lipotrichesfervida* (Smith, 1875), female **A** dorsal habitus **B** lateral habitus.

**Female** (Fig. 3). Body length 7 mm. ***Head***: Coarsely pitted vertex, slightly convex clypeus that is anteriorly rounded, antennae fulvous, ocelli without the bead; clypeus, gena, and area near the ocellus covered with very short, dense, pale fulvous pubescence. ***Mesosoma***: Closely and finely punctured, posteriorly truncate, slightly compressed sides, the space at the base moderately wide, coarsely reticulate; femora and tibiae incrassate and swollen, ventral femora, tarsi, and tibiae black, tibial bristles of hind legs with long branches and short rachises, wings slightly fuscous and hyaline; a fine line over the tegulae, sides of the median segment, thorax, and postscutellum; a broad transverse band on the mesonotum anteriorly covered with very short, dense pale fulvous pubescence. ***Metasoma***: Closely and finely punctured, broad transverse bands on the apical margins of the terga 1–6, metasomal segments covered with very short, dense pale fulvous pubescence, fairly fine superficial punctation on tergum 1.

##### Distribution.

Lipotriches (Armatriches) fervida is distributed in Pakistan, India, and Sri Lanka (Pauly 2009; Saini and Rathor 2012; Pauly 2014; Majumder et al. 2020; Ascher and Pickering 2024).

##### Observed floral host.

*Sorghumhalepense*.

#### ﻿ Austronomia pilipes

Taxon classificationAnimaliaHymenopteraHalictidae

(Smith, 1875)

EBEA7DD8-EC44-53E1-BA73-E865B262DE87

Figs 2A, B , 4

Nomia pilipes Smith, 1875.

##### Material examined.

Pakistan. Punjab, Bahawalpur • 1♀, 27 October 2020, A. Ahmad • 7♀ 5♂, 18 November 2020, W. Akram & A. Ahmad • 1♂, 23 November 2020, A. Sajjad.

##### Diagnosis.

**Figure 4.**
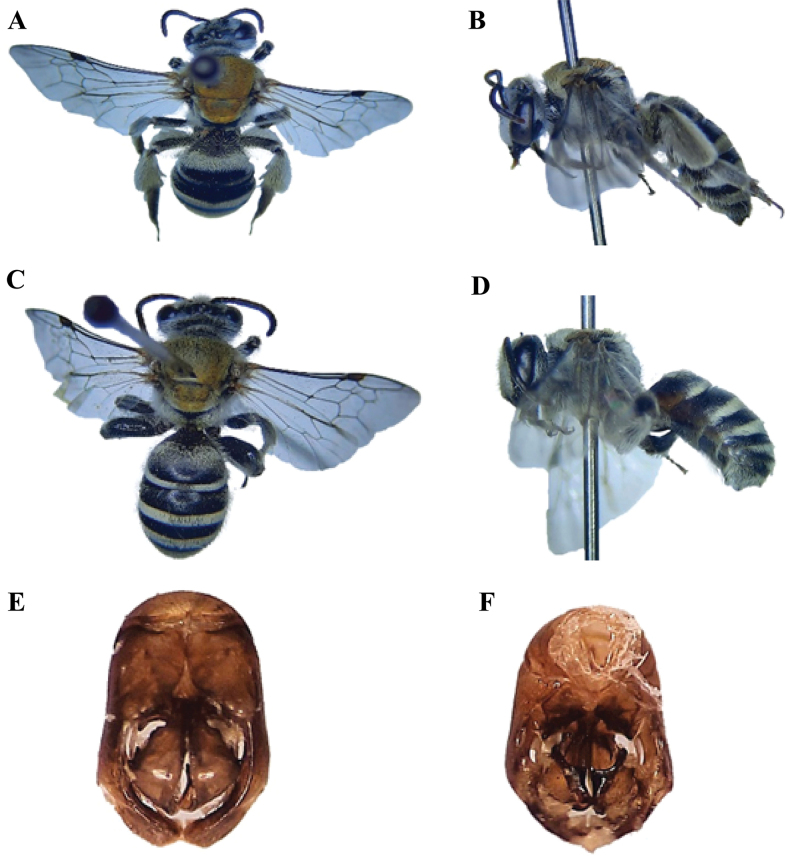
*Austronomiapilipes* (Smith, 1875) **A** dorsal habitus, female **B** lateral habitus, female **C** dorsal habitus, male **D** lateral habitus, male **E** male genitalia, dorsal view **F** male genitalia, ventral view.

**Female** (Fig. 4A, B): Body length ranges from 10–12 mm. **Male** (Fig. 4C, D): Body length ranges from 10–11 mm. ***Head***: Closely punctured and densely pubescent, antennae black, longitudinal carina absent on the clypeus, supraclypeal area and clypeus normally convex, head covered with glittering white pubescence. ***Mesosoma***: Closely punctured and densely pubescent, truncate, posteriorly pubescent, bare sides; in the middle, smooth, nearly impunctate; the space at the base very coarsely punctured and concave; scutum, scutellum, and post-scutellum covered with dense beige scaly pubescence, testaceous tegulae. Wings hyaline with testaceous veins. Sides of the mesosoma covered with glittering white pubescence, abundant glittering white scopa on the hind legs. ***Metasoma***: Slightly shiny, convex, and broad with a few dispersed punctures at the bases of the segments, terga 1–5 with glittering white pubescent apical bands. ***Genitalia*** (Fig. 4E, F): Genital gonobase small and robust, gonocoxite wider at the base and becoming narrower at the apex, gonostylus slender, and penis valves wider in the middle.

##### Distribution.

Lipotriches (Austronomia) pilipes is only distributed in Pakistan and India (Pauly 2009; Gupta 2013; Ascher and Pickering 2024).

##### Observed floral hosts.

*Salsolabaryosma*, *Tamarixaphylla*, and *Daturafastuosa*.

#### ﻿ Lipotriches (Lipotriches) fulvinerva

Taxon classificationAnimaliaHymenopteraHalictidae

(Cameron, 1907)

403C32DE-82EB-505C-A207-F9AEB8FE0CAC

Figs 2F–H , 5

Nomia fulvinerva Cameron, 1907.

##### Material examined.

Pakistan. Punjab, Bahawalpur • 1♀, 17 October 2020, W. Akram.

##### Diagnosis.

**Figure 5.**
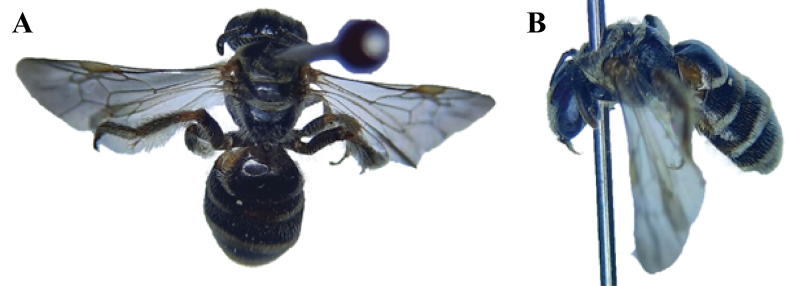
*Lipotrichesfulvinerva* (Cameron, 1907), female **A** dorsal habitus **B** lateral habitus.

**Female** (Fig. 5): Body length ranges from 6–7 mm. ***Head***: Coarsely pitted vertex, slightly convex clypeus and anteriorly rounded, antennae fulvous, scape with pale pubescence, clypeus, gena, and the area near the ocellus covered with very short fulvous pubescence, mandibles dark red. ***Mesosoma***: Closely and finely punctured, posteriorly truncate, somewhat compressed sides, the space at the base moderately wide, coarsely reticulate, tibiae and tarsi completely orange, tibial bristles of hind legs with long branches and short rachises, wings slightly fuscous and hyaline, a fine line over the tegulae and a broad transverse band on the mesonotum anteriorly covered with very short, dense pale fulvous pubescence. ***Metasoma***: Broad transverse bands on the apical or distal margins of the terga 1–6 abdominal segments that are covered with a very short and dense white pubescence, tergum 1 with matte punctures.

##### Distribution.

Lipotriches (Lipotriches) fulvinerva is distributed in India, Myanmar, Pakistan, and Sri Lanka (Inoka et al. 2006; Pauly 2009; Saini and Rathor 2012; Gupta 2013; Pauly 2014; Sharma et al. 2016; Saini et al. 2018; Majumder et al. 2020; Ascher and Pickering 2024).

##### Observed floral host.

*Pennisetumglaucum*.

### ﻿Key to species of *Nomia* Letreille, 1804

**Table:** 

1	Basal vein in forewing slightly arched (Fig. 6A); abdominal terga with white bands (Fig. 6B); femora, tibiae, and tarsi black (Fig. 6C)	***N.interstitialis***
–	Basal vein in forewing not arched; abdominal terga without white bands; femora, tibiae, and tarsi not black	**2**
2	Tegulae testaceous (Fig. 6D); wings apically fuscous; abdominal terga with yellow bands, shiny and smooth (Fig. 6E); sternum black; femora, tibiae, and tarsi in males yellow (Fig. 6F)	***N.curvipes***
–	Tegulae partially whitish (Fig. 6G); wings apically not fuscous; abdominal terga having green bands with reddish blue effulgence, shiny and smooth (Fig. 6H); sternum testaceous; femora and tibiae in males orange-red (Fig. 6I)	***N.westwoodi***

#### ﻿ Nomia (Nomia) curvipes

Taxon classificationAnimaliaHymenopteraHalictidae

(Fabricius, 1793)

021D98A9-E2B4-54B8-AE1F-F043E5097524

Figs 6D–F , 7

Andrena curvipes Fabricius, 1793.

##### Material examined.

Pakistan. Punjab, Bahawalpur • 1♂, 26 May 2020, A. Ahmad • 1♂, 6 June 2020, W. Akram • 1♀, 21 June 2020, W. Akram • 1♀ 2♂, 27 June 2020, W. Akram & A. Sajjad • 2♂, 28 June 2020, A. Ahmad • 2♀ 3♂, 4 July 2020 • 2♀ 1♂, 11 July 2020, A. Sajjad & A. Ahmad • 3♀ 6♂, 13 July 2020, W. Akram, A. Sajjad & A. Ahmad • 4♀, 20 July 2020, W. Akram & A. Ahmad • 1♀, 21 July 2020, A. Sajjad • 1♂, 6 August viii.2020, W. Akram • 1♀ 1♂, 8 August 2020, W. Akram & A. Ahmad; 1♀, 21 September 2020, W. Akram. Lodhran • 1♀ 1♂, 27 July 2020, W. Akram & A. Ahmad • 1♀, 20 July 2020, W. Akram • 2♀, 21 July 2020, W. Akram. Multan • 1♂, 27 June 2020, M.K. Rafique • 1♀, 20 August 2020, A. Sajjad • 1♂, 27 August 2020, M.K. Rafique. Khanewal • 3♂, 15 July 2020, A. Sajjad • 1♀ 2♂, 18 September 2020, A. Sajjad & W. Akram. Rahimyar Khan • 1♂, 21 July 2020, S. Hussain • 2♀, 8 August 2020, S. Hussain & Z.H. Dahri • 1♀ 1♂, 4 October 2020, Z.H. Dahri.

**Figure 6.**
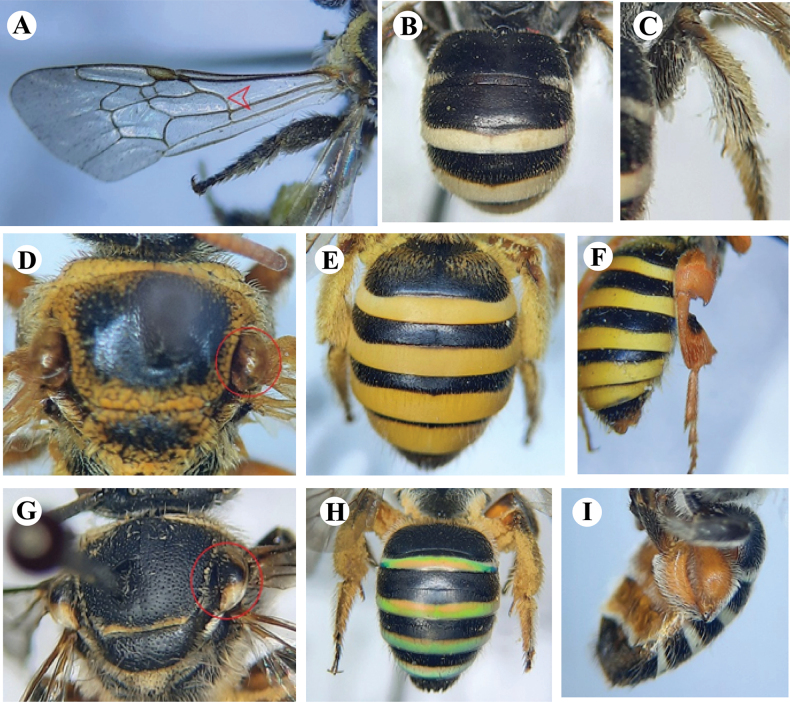
**A–C***Nomiainterstitialis* Cameron, 1898, female **A** left forewing showing basal vein **B** metasoma **C** hind leg **D–F***N.curvipes* (Fabricius, 1793), **D** mesosoma showing tegulae of female **E** metasoma of female showing terga **F** hind leg of male **G–I***N.westwoodi* (Gribodo, 1894) **G** mesosoma showing tegulae of female **H** metasoma of female showing terga **I** hind leg of male.

##### Diagnosis.

**Figure 7.**
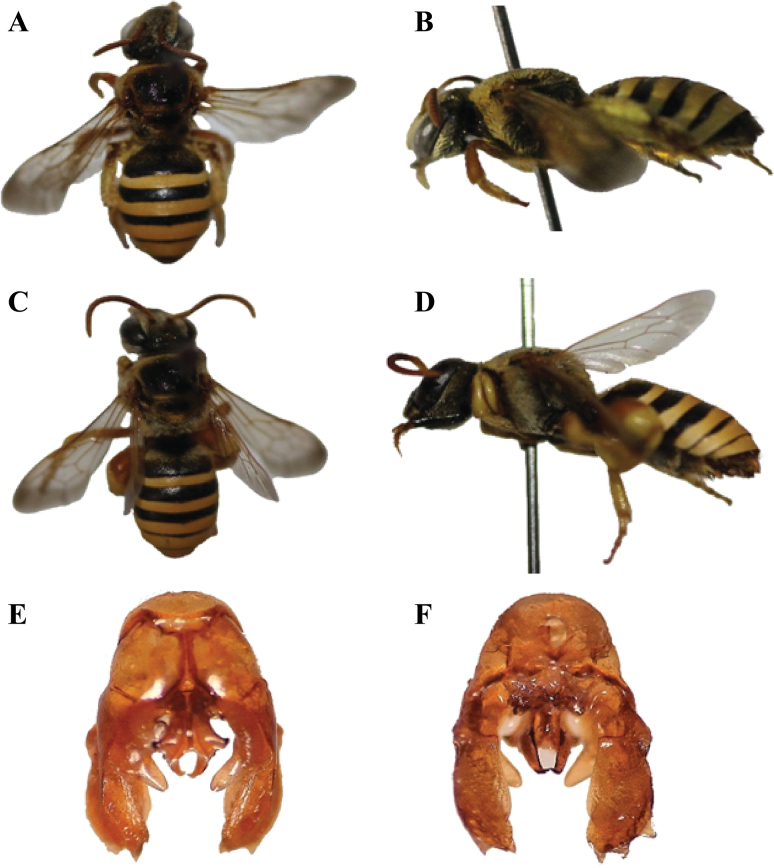
*Nomiacurvipes* (Fabricius, 1793) **A** dorsal habitus, female **B** lateral habitus, female **C** dorsal habitus, male **D** lateral habitus, male **E** male genitalia, dorsal view **F** male genitalia, ventral view.

**Female** (Fig. 7A, B): Body length ranges from 10–12 mm. ***Head***: Closely but lightly punctate, clypeus transverse anteriorly and slightly convex, antennae testaceous brown. ***Mesosoma***: Dense but coarsely punctured, rounded at the sides, concavo-truncate at the apex, testaceous brown legs, hind legs covered with glittering pale pubescence, tegulae testaceous brown, median segment and mesosoma with dense fulvo-ferruginous pubescence, fulvo-hyaline wings, slightly fuscous at apical margin, veins pale. ***Metasoma***: Closely but lightly punctured, the apical margins of metasomal segments 1–4 with bright yellow bands, shiny and smooth. **Male** (Fig. 7C, D): Body length ranges from 10–11 mm, hind femur very swollen, hind tibia broadened from the inner side; coxae, trochanters, and base of the femora black, femora, tibiae, and tarsi yellow. ***Genitalia*** (Fig. 7E, F): Genital dorsal gonocoxite teeth prominent but short and slender penis valves wider in the middle and hook-like at the apex.

##### Distribution.

Nomia (Nomia) curvipes is distributed in India, Nepal, Pakistan, and Sri Lanka (Pauly 2008, 2009; Wijesekara 2001; Saini and Rathor 2012; Sharma et al. 2016; Chandra et al. 2019; Ascher and Pickering 2024). In Pakistan, this species has been reported from Layyah, Attock, Chakwal, Jhelum, Islamabad, and Rawalpindi (Bodlah et al. 2016; Aslam et al. 2020; Bodlah et al. 2020).

##### Observed floral hosts.

*Luffaacutangula*, *Tribulusterrestris*, *Cucumismomordica*, *Citrulluslanatus*, *Convolvulusarvensis*, *Portulacaoleracea*, *Cucumismelo*, *Tamarixaphylla*, *Medicagosativa*, and *Capsicumfrutescens*. Some other floral hosts i.e., *Carthamusoxycantha* and *Phaseolusvulgaris* of *N.curvipes* have been reported (Bodlah et al. 2020).

#### ﻿ Nomia (Hoplonomia) westwoodi

Taxon classificationAnimaliaHymenopteraHalictidae

(Gribodo, 1894)

45B88E40-2DB7-5AA6-8A46-35D4C0E159A5

Figs 6G–I , 8

Nomia westwoodi Gribodo, 1894.

##### Material examined.

Pakistan. Punjab, Bahawalpur • 1♀ 2♂, 5 June 2020, W. Akram & A. Sajjad • 2♂, 6 July 2020, A. Sajjad • 1♂, 27 August 2020, A. Ahmad • 2♀, 8 October 2020, W. Akram • 1♂, 17 October 2020, W. Akram. Lodhran • 2♂, 6 September 2020, A. Ahmad & W. Akram. Multan • 2♂, 21 June 2020, A. Sajjad & M.K. Rafique • 1♂, 30 March 2021, A. Sajjad. Khanewal • 2♂, 29 March 2021, W. Akram & A. Ahmad. Rahimyar Khan • 3♀ 3♂, 4 October 2020, Z.H. Dahri & S. Hussain • 6♀, 5 October 2020, Z.H. Dahri & S. Hussain.

##### Diagnosis.

**Figure 8.**
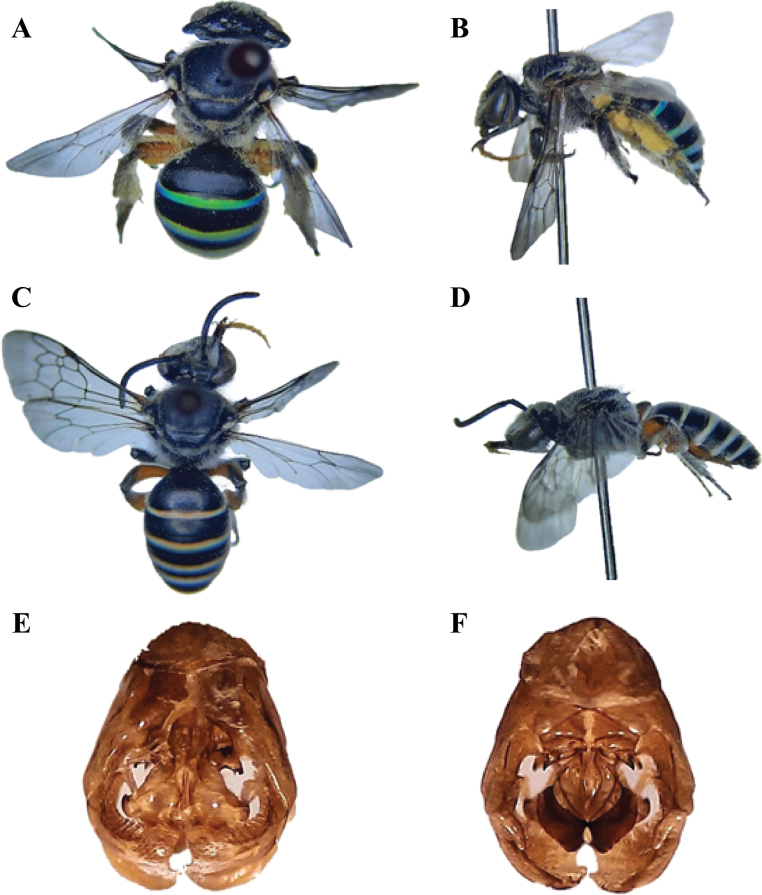
*Nomiawestwoodi* (Gribodo, 1894) **A** dorsal habitus, female **B** lateral habitus, female **C** dorsal habitus, male **D** lateral habitus, male **E** male genitalia, dorsal view **F** male genitalia, ventral view.

**Female** (Fig. 8A, B): Length of the body ranges from 8–12 mm. ***Head***: Transverse head with pale pubescence, antennae black, the apex of the mandibles with two teeth, inner much shorter than outer, clypeus not produced, 6-segmented maxillary palpi, 4-segmented labial palpi. ***Mesosoma***: Black, coarsely punctured, femur and hind tibia not swollen; coxa, trochanter, and femur orange-red, tibia and tarsi black, whitish pale pubescence on hind legs, wings fuscous hyaline with rounded radial cell at the apex, three submarginal cells, 3^rd^ longest or as long as 1^st^. ***Metasoma***: Tergum 1–4 of metasoma with apical shiny greenish bands, sternum testaceous. **Male** (Fig. 8C, D): Body length 7–9 mm, the inner angle of the apex of the tibiae produced and rounded, posterior legs and metasoma beneath testaceous. ***Genitalia*** (Fig. 8E, F): Genital gonobase prominent, gonostylus apically wider, curled inward and the inner side equipped with comb-like structure, penis valves wider in the middle.

##### Distribution.

Nomia (Hoplonomia) westwoodi is distributed in Afghanistan, India, Pakistan, and Sri Lanka (Wijesekara 2001; Inoka et al. 2006; Pauly 2009; Saini and Rathor 2012; Sharma et al. 2016; Ascher and Pickering 2024). In Pakistan, this species has been reported from Layyah, Chakwal, and Islamabad (Bodlah et al. 2016; Aslam et al. 2020; Bodlah et al. 2020).

##### Observed floral hosts.

*Parkinsoniaaculeata*, *Punicagranatum*, *Cajanuscajan*, *Corchorusolitorius*, *Erucasativa*, *Arachishypogaea*, *Lagerstroemiaindica* and *Luffaacutangula*. Some other floral hosts of *N.westwoodi*, i.e., *Lablabpurpureus*, *Solanummelongena*, *Muntingiacalabura*, *Vignaradiata*, and *Vigna* sp. have been reported (Inoka et al. 2006).

#### ﻿ Nomia (Leuconomia) interstitialis

Taxon classificationAnimaliaHymenopteraHalictidae

Cameron, 1898

9FD24759-3FE7-51BF-BA36-E757140B441F

Figs 6A–C , 9

Nomia rothneyi Cameron, 1904; Leuconomiainterstitialis (Cameron, 1898).

##### Material examined.

Pakistan. Punjab, Bahawalpur • 1♀, 28 June 2020, W. Akram • 1♀, 27 July 2020, W. Akram • 1♀ 1♂, 25 August 2020, W. Akram • 1♀, 7 October 2020, A. Ahmad; Lodhran • 1♀, 21 July 2020, W. Akram; Multan • 1♂, 8 August 2020, A. Sajjad; Rahimyar Khan • 2♀ 4♂, 4 October 2020, Z.H. Dahri & S. Hussain.

##### Diagnosis.

**Figure 9.**
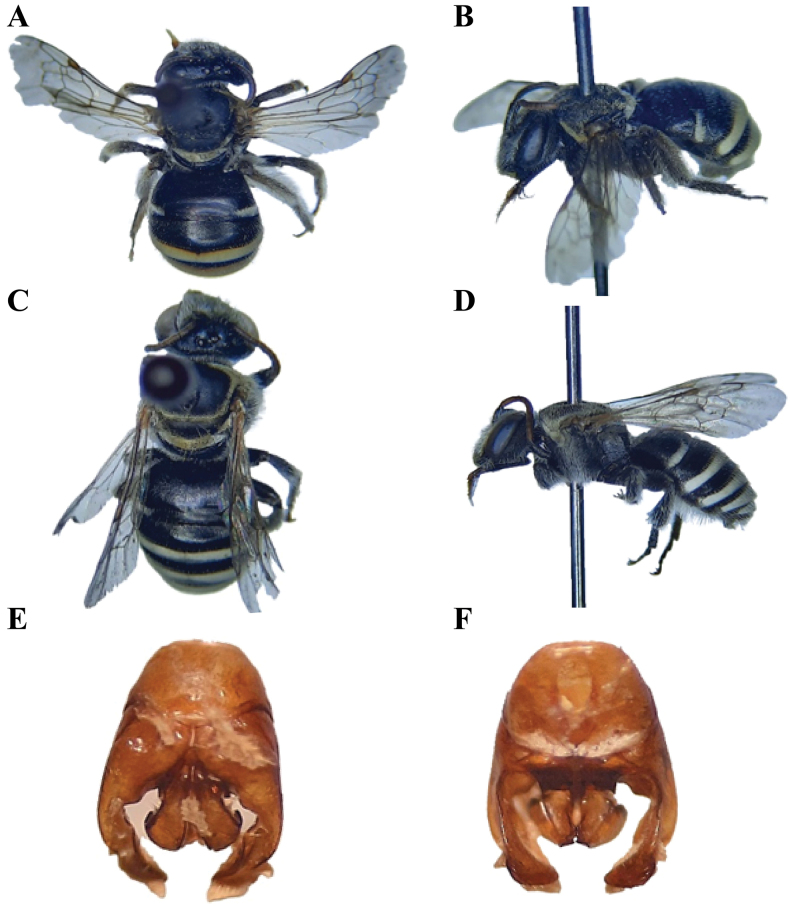
*Nomiainterstitialis* Cameron, 1898, **A** dorsal habitus, female **B** lateral habitus, female **C** dorsal habitus, male **D** lateral habitus, male **E** male genitalia, dorsal view **F** male genitalia, ventral view.

**Female** (Fig. 9A, B): Body length 7–8 mm. ***Mesosoma***: Scutum glabrous, with felted fringe only around periphery, scutellum double-humped. ***Metasoma***: All terga black, enamel-like bands occupy almost the entire apical depression of the terga, tergum 1 without enamel band but with pubescent lateral fringes, tergum 1 completely matte satin with a few very shallow and spaced punctures. **Male** (Fig. 9C, D): Body length 6–7 mm. ***Mesosoma***: Scutum with fine and dense punctation, matte shagreened, glabrous except the periphery with a felted fringe; propodeum rounded, matte, the propodeal area triangular; double-humped scutellum. ***Metasoma***: Sternum 3 without structures, with apical margin very slightly emarginated in its center; sternum 5 with two wide horizontal laminated structures; sternum 6 slightly wavy; sterna 2–4 with long bristles on the side parts. Tergum 1 without enamel bands but with a silky fringe on each side; hind femur and hind tibia black, not very developed. ***Genitalia*** (Fig. 9E, F): Genital gonobase more prominent, gonostylus apically narrower and with a bunch of hairs, penis valves wider than longer.

##### Distribution.

Nomia (Leuconomia) interstitialis is distributed in India and Pakistan (Pauly 2009; Saini and Rathor 2012; Gupta 2013; Chandra et al. 2019; Ascher and Pickering 2024). This species is reported for the first time from southern Punjab, Pakistan.

##### Observed floral hosts.

*Luffaacutangula*, *Portulacaoleracea*, *Cucumismomordica*, and *Cajanuscajan*.

### ﻿Key to species of *Pseudapis* Kirby, 1900

**Table:** 

1	Antennae testaceous; head in lateral view showing carinae under the compound eye (Fig. 10A); propodeum reddish (Fig. 10B); tergum 1 completely and tergum 2 partially reddish; femora, tibiae, and tarsi orange with pale scopa (Fig. 10C)	***P.nilotica***
–	Antennae black; carinae under the compound eye absent; propodeum black; abdominal terga black; femora, tibiae, and tarsi black or testaceous with white scopa	**2**
2	Tegulae orange (Fig. 10D); femora, tibiae, and tarsi black in both sexes (Fig. 10E); terga with basal hair bands and without apical shiny golden layer (Fig. 10F)	***P.oxybeloides***
–	Tegulae posteriorly hyaline (Fig. 10G); in females, femora black, tibiae and tarsi testaceous whereas in males basitarsi yellowish and shiny (Fig. 10H); terga with basal hair bands and apical shiny golden layer (Fig. 10I)	***P.bispinosa***

#### ﻿ Pseudapis (Pseudapis) oxybeloides

Taxon classificationAnimaliaHymenopteraHalictidae

(Smith, 1875)

413CB1B2-16AF-55BD-A4DF-82F736755305

Figs 10D–F , 11

Nomia oxybeloides Smith, 1875.

##### Material examined.

Pakistan. Punjab, Bahawalpur • 1♀, 12 April 2021, W. Akram • 6♀ 2♂, 5 June 2020, A. Sajjad, W. Akram & A. Ahmad • 2♀ 1♂, 27 June 2020, A. Ahmad • 3♀ 3♂, 20 July 2020, A. Sajjad, W. Akram & A. Ahmad • 1♀, 21 July 2020, A. Ahmad • 8♀ 7♂, 8 August 2020, W. Akram, A. Sajjad & A. Ahmad; 1♀, 18 September 2020, A. Sajjad • 1♀, 24 November 2020, W. Akram; Lodhran • 2♀, 15 June 2020, A. Ahmad • 1♀ 1♂, 18 June 2020, W. Akram • 1♀, 2 August 2020, W. Akram; Multan • 3♀ 1♂, 16 June 2020, M.K. Rafique & A. Sajjad • 1♀, 18 June 2020, A. Sajjad • 2♀ 3♂, 2 August 2020, M.K. Rafique & A. Sajjad • 2♀, 3 August 2020, A. Sajjad; Khanewal • 3♀, 19 June 2020, W. Akram & A. Ahmad • 2♀ 1♂, 20 June 2020, W. Akram & A. Ahmad • 1♀, 4 July 2020, A. Sajjad; Rahimyar Khan • 3♀, 12 June 2020, W. Akram & A. Ahmad • 1♀, 25 August 2020, Z.H. Dahri; Dera Ghazi Khan • 2♀ 1♂, 27 August 2020, A. Sajjad, W. Akram & A. Ahmad.

**Figure 10.**
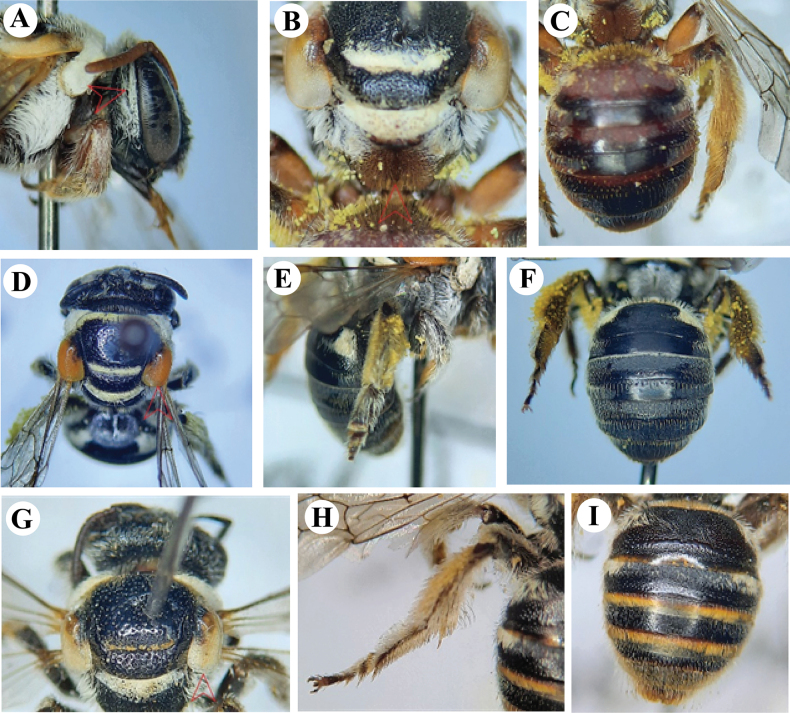
**A–C***Pseudapisnilotica* (Smith, 1875), female **A** lateral view of head showing carinae under the compound eye **B** mesosoma showing propodeum **C** metasoma showing terga **D–F***P.oxybeloides* (Smith, 1875), female **D** head and mesosoma showing tegulae **E** hind leg **F** metasoma showing terga **G–I***P.bispinosa* (Brullé, 1832), female **G** mesosoma showing tegulae **H** hind leg **I** metasoma showing terga.

##### Diagnosis.

**Figure 11.**
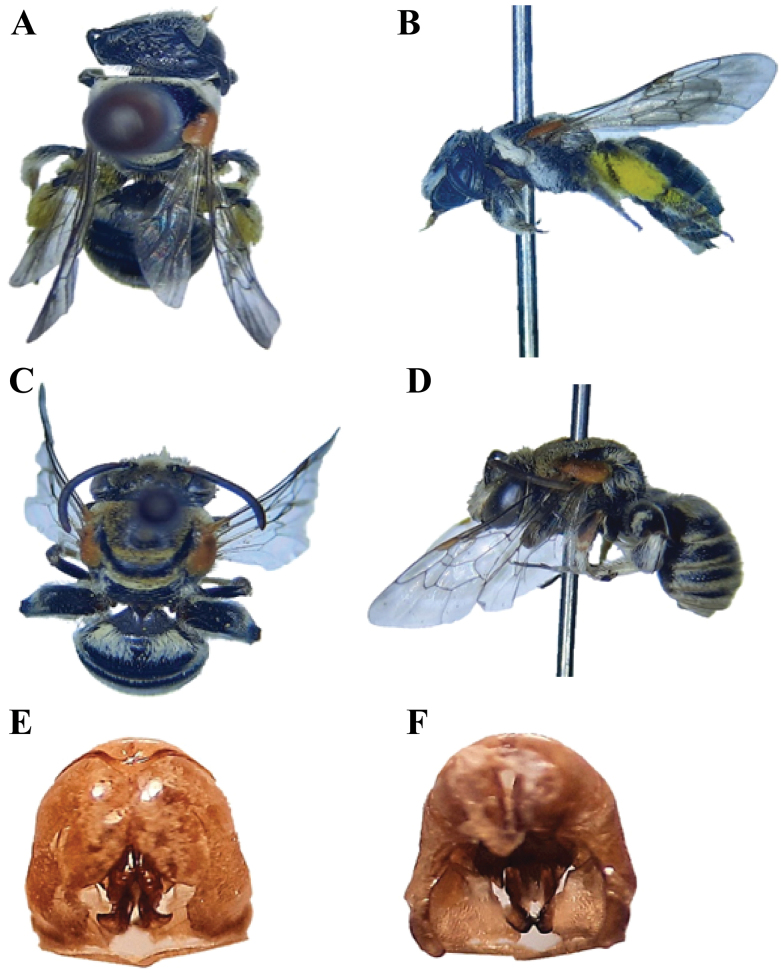
*Pseudapisoxybeloides* (Smith, 1875) **A** dorsal habitus, female **B** lateral habitus, female **C** dorsal habitus, male **D** lateral habitus, male **E** male genitalia, dorsal view **F** male genitalia, ventral view.

**Female** (Fig. 11A, B): ***Head***: Densely and finely punctured, clypeus slightly flat and arched from anterior side, the head and clypeus in front with pubescence of shiny silvery white. ***Mesosoma***: Coarsely and lightly punctured, tegulae very large, broader than long and testaceous, propodeum curved at the sides, sharply truncated at the posterior side and more finely punctured as compared to mesonotum, basal space very narrow, with only a row of deep rough punctures; the legs on the outside, post-scutellum, and a line on pronotum with pubescence of shiny silvery white, the mesosoma often with fulvous pubescence. ***Metasoma***: Shiny, basal three metasomal segments dorsally with an unclear diagonally impressed line, margins of metasomal segments with pubescence of shiny silvery white. **Male** (Fig. 11C, D): Similar but smaller with a fulvous shade of pubescence, hind tibiae and femora swollen, the lower angle of each tibia from the apex produced into a long flat process with testaceous white, externally curved, the inner margin straight, hyaline wings in both sexes, tegulae and veins testaceous. ***Genitalia*** (Fig. 11E, F): Genital gonocoxite robust and gonostylus lamellate.

##### Distribution.

Pseudapis (Pseudapis) oxybeloides is distributed in Bangladesh, India, Pakistan, and Sri Lanka (Inoka et al. 2006; Ascher and Rasmussen 2010; Saini and Rathor 2012; Gupta 2013; Sharma et al. 2016; Chandra et al. 2019; Ascher and Pickering 2024). This species has already been reported from Multan, southern Punjab (Sajjad et al. 2019b).

##### Observed floral hosts.

*Luffaacutangula*, *Launaeanudicaulis*, *Cucumismomordica*, *Trianthemaportulacastrum*, *Capsicumfrutescens*, *Convolvulusarvensis*, *Corchorusolitorius*, *Lagenariasiceraria*, *Raphanussativus*, *Jatrophaintegerrima*, *Solanumvirginianum*, and *Salsolabaryosma*. Some other floral hosts i.e., *Cucumissativus*, *Desmodiumheterophyllum*, *Hedyotiscorymbose*, *Ipomoeamauritiana*, and *Vernoniacinerea* of *P.oxybeloides* have been reported (Inoka et al. 2006).

#### ﻿ Pseudapis (Nomiapis) bispinosa

Taxon classificationAnimaliaHymenopteraHalictidae

(Brullé, 1832)

AA1F82CA-1076-5ACD-A2FA-BDECC1FF3F54

Figs 10G–I , 12

Nomia bispinosa Brullé, 1832.

##### Material examined.

Pakistan. Punjab, Bahawalpur • 8♀ 7♂, 9 April 2020, W. Akram, A. Sajjad & A. Ahmad • 1♂, 11 April 2020, W. Akram • 1♀ 8♂, 14 April 2020, W. Akram, A. Sajjad & A. Ahmad • 2♀ 2♂, 29 May 2020, W. Akram & A. Ahmad • 1♀ 1♂, 18 September 2020, W. Akram; Khanewal • 6♀ 13♂, 31 March 2021, A. Sajjad, W. Akram & A. Ahmad; Multan • 2♀ 1♂, 29 March 2020, A. Sajjad & M.K. Rafique • 1♀ 2♂, 30 March 2020, A. Sajjad.

##### Diagnosis.

**Figure 12.**
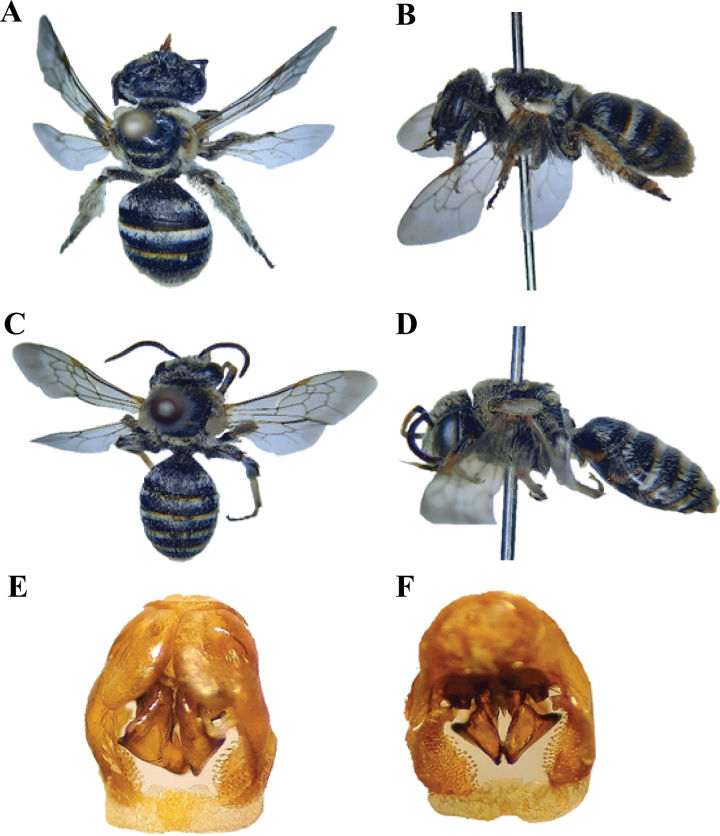
*Pseudapisbispinosa* (Brullé, 1832) **A** dorsal habitus, female **B** lateral habitus, female **C** dorsal habitus, male **D** lateral habitus, male **E** male genitalia, dorsal view **F** male genitalia, ventral view.

**Female** (Fig. 12A, B): Body length 9.5–12 mm. ***Head***: Clypeus narrower ~ 2× wider than the longer, frontal line wide and prominent, vertex ~ 3× diameter of ocellus. ***Mesosoma***: Tegulae posteriorly hyaline; femora black, tibiae and tarsi testaceous, mesoscutum with semi-recumbent scaly hairs along with anterior pronotum. ***Metasoma***: Abdominal terga with basal hair bands and apical shiny golden layer. **Male** (Fig. 12C, D): Body length 9–11.5 mm. Mesothorax without any process on the ventral surface, scarcely developed distal process of hind tibia, basitarsi yellowish and shiny, sternum 4 with straight posterior margin, thickening in the middle and without emargination. ***Genitalia*** (Fig. 12E, F): Similar to *P.oxybeloides* but the inner side of the gonostylus contains comb-like structure.

##### Distribution.

Pseudapis (Nomiapis) bispinosa is distributed in Afghanistan, Algeria, Egypt, India, Iran, Iraq, Italy, China, Cyprus, Georgia, Greece, Hungary, Kazakhstan, Malta, Morocco, Pakistan, Russia, Spain, Tunisia, Turkey, Turkmenistan, and Ukraine (Astafurova and Pesenko 2006; Ascher and Rasmussen 2010; Güler et al. 2011; Saini and Rathor 2012; Dunford et al. 2014; Safi et al. 2016; Augul 2018; Hosseini et al. 2019; Nazari et al. 2019; Lhomme et al. 2020; Ascher and Pickering 2024). This species is the first time reported from southern Punjab, Pakistan.

##### Observed floral hosts.

*Tageteserecta*, *Heliotropiumcrispum*, *Chrozophoratinctoria*, *Trifoliumalexandrinum*, *Calotropisprocera*, *Carthamustinctorius*, *Carthamusoxyacantha*, and *Convolvulusarvensis*. Some other floral hosts i.e., *Medicago* sp. and *Mentha* sp. of *P.bispinosa* have been reported (Nazari et al. 2019).

#### ﻿ Pseudapis (Pseudapis) nilotica

Taxon classificationAnimaliaHymenopteraHalictidae

(Smith, 1875)

CAE3AC9C-5267-5500-9F4E-73E93561C16A

Figs 10A–C , 13

Nomia nilotica Smith, 1875.

##### Material examined.

Bahawalpur • 5♀ 5♂, 4 July 2020, W. Akram & A. Ahmad • 1♀ 2♂, 11 July 2020, W. Akram & A. Ahmad • 2♀ 4♂, 5 September 2020, W. Akram & A. Ahmad • 1♂, 26 October 2020, W. Akram.

##### Diagnosis.

**Figure 13.**
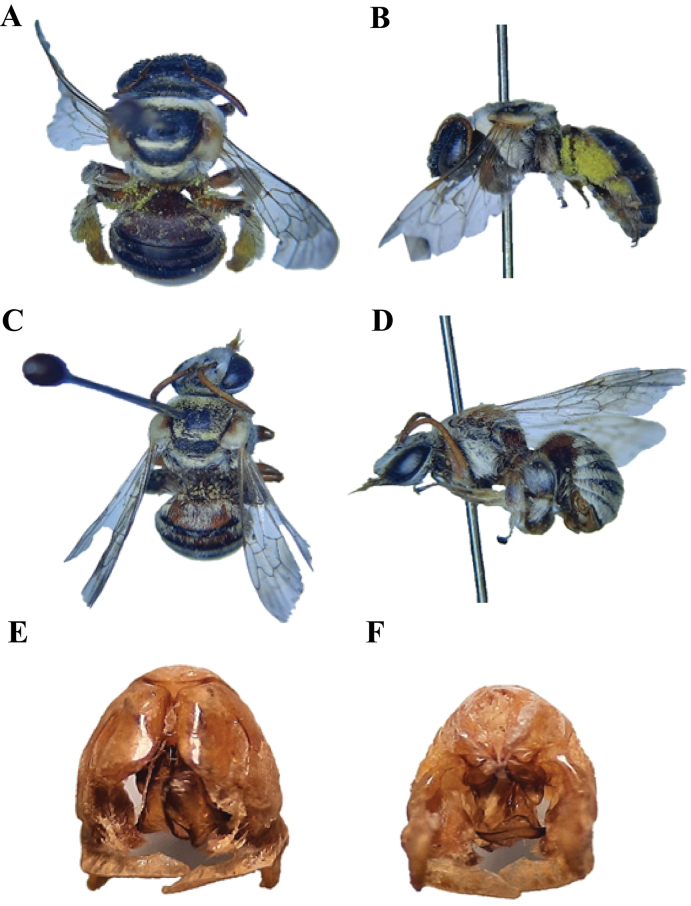
*Pseudapisnilotica* (Smith, 1875) **A** dorsal habitus, female **B** lateral habitus, female **C** dorsal habitus, male **D** lateral habitus, male **E** male genitalia, dorsal view **F** male genitalia, ventral view.

**Female** (Fig. 13A, B): ***Head***: Lateral view showing carinae under the compound eye, antennae testaceous. ***Mesosoma***: Propodeum reddish, femora, tibiae and tarsi orange with pale scopa. ***Metasoma***: Tergum 1 completely and tergum 2 partially reddish, S4 medioapically with a U-shaped depression bounded by two parallel callosities which arise from an extension of the disc above the marginal area, these callosities prolonged as short, blunt teeth, the emargination between them shallow; the depressed marginal area narrow, beneath the discal teeth and nearly concealed by them with two further small blunt teeth; the marginal teeth very small and nearly concealed beneath the upper teeth. **Male** (Fig. 13C, D): Antennae testaceous, scaly hairs on the dorsal mesosoma, propodeum reddish, anterior margin of the anterior lobe of hind leg tibia angularly emarginate, metasomal tergites with white hair bands. ***Genitalia*** (Fig. 13E, F): Similar to *P.oxybeloides* but the gonostylus contains fringe of hairs.

##### Distribution.

Pseudapis (Pseudapis) nilotica is distributed in Afghanistan, Algeria, Djibouti, Egypt, Ethiopia, Iran, Iraq, Jordan, Libya, Morocco, Niger, Pakistan, Russia, Saudi Arabia, Sudan, Turkmenistan, and United Arab Emirates (Astafurova and Pesenko 2006; Ascher and Rasmussen 2010; Khodaparast and Monfared 2012; Shebl et al. 2013; Dunford et al. 2014; Augul 2018; Hosseini et al. 2019; Lhomme et al. 2020; Ascher and Pickering 2024). This species is the first time reported from the southern Punjab, Pakistan.

##### Observed floral host.

*Chrozophoratinctoria* and *Citrulluscolocynthis*. Some other floral hosts i.e., *Vitexagnus-castus* and *Pelargonium* sp. of *P.nilotica* have been reported (Khodaparast and Monfared 2012).

## ﻿Conclusions

Eight species of Nomiinae bees are reported for the first time from southern Punjab, Pakistan. Brief descriptions of the genitalia are also provided for each species except Lipotriches (Armatriches) fervida and Lipotriches (Lipotriches) fulvinerva, as male specimens were not found throughout the study period. Although the number of species is not large, 251 specimens were found in total. More studies are needed to further explore the numbers of genera and species of the subfamily Nomiinae from this region.

## Supplementary Material

XML Treatment for Lipotriches (Armatriches) fervida

XML Treatment for Austronomia pilipes

XML Treatment for Lipotriches (Lipotriches) fulvinerva

XML Treatment for Nomia (Nomia) curvipes

XML Treatment for Nomia (Hoplonomia) westwoodi

XML Treatment for Nomia (Leuconomia) interstitialis

XML Treatment for Pseudapis (Pseudapis) oxybeloides

XML Treatment for Pseudapis (Nomiapis) bispinosa

XML Treatment for Pseudapis (Pseudapis) nilotica

## ﻿Additional information

### Author ORCIDs

Huanhuan Chen https://orcid.org/0000-0001-9477-1014

Waseem Akram https://orcid.org/0000-0001-8478-2588

Muhammad Naeem https://orcid.org/0000-0001-6720-5463

Nawaz Haider Bashir https://orcid.org/0000-0003-0206-2435

Sabir Hussain https://orcid.org/0000-0002-3532-0907

Maryam Riasat https://orcid.org/0009-0003-2884-6665

Asif Sajjad https://orcid.org/0000-0002-4246-1484

Ruilin Tian https://orcid.org/0000-0002-6005-4014

Ammad Ahmad https://orcid.org/0000-0003-1975-5609

Muhammad Khalid Rafique https://orcid.org/0000-0003-3612-9765

Zamin Hussain Dahri https://orcid.org/0009-0002-9634-4379

### Data availability

All of the data that support the findings of this study are available in the main text.
